# Macular buckling combined with autologous retinal transplantation for the treatment of recurrent macular hole retinal detachment: A case report

**DOI:** 10.1097/MD.0000000000042621

**Published:** 2025-05-30

**Authors:** Rongrong Zhang, Mingxing Li, Jie Li, Dan Xia, Yu Li, Jiahao Li, Wenqing Deng, Chenyu Yang, Jing Wang, Jiayu Ma, Yingjun Li

**Affiliations:** a Department of Ophthalmology, Fuyang People’s Hospital Affiliated to Anhui Medical University, Fuyang, Anhui Province, China; b Department of Ophthalmology, The Affiliated Xuzhou Municipal Hospital of Xuzhou Medical University, Xuzhou, Jiangsu Province, China; c Department of Bengbu Medical University, Bengbu, Anhui Province, China; d Department of Ophthalmology, Putuo District People’s Hospital of Zhoushan City, Zhoushan, Zhejiang Province, China; e Department of Anhui Medical University, Hefei, Anhui Province, China.

**Keywords:** autologous retinal transplant, highly myopia, macular buckle, macular hole retinal detachment, posterior staphyloma, recurrent

## Abstract

**Rationale:**

Recurrent macular hole retinal detachment (MHRD) in high myopia is a challenging clinical problem with limited therapeutic options. Traditional treatments, such as pars plana vitrectomy (PPV) and internal limiting membrane (ILM) peeling, often fail to achieve satisfactory outcomes in these complex cases. This study aims to report a novel surgical approach involving macular buckling and autologous retinal transplantation for the treatment of recurrent MHRD in high myopia, highlighting its potential to improve anatomical and functional outcomes.

**Patient concerns:**

A 63-year-old female patient presented with decreased vision in her left eye, 2 months after the removal of silicone oil (SO). She had previously undergone PPV, ILM peeling, and SO tamponade in the same eye. Her best corrected visual acuity (BCVA) was hand movement, and axial length was 30.81 mm. Optical coherence tomography (OCT) revealed retinal detachment involving the posterior pole with a macular hole.

**Diagnoses:**

Recurrent macular hole retinal detachment in the left eye was diagnosed.

**Interventions:**

Given concerns about insufficient residual ILM, the patient underwent a combined surgical procedure, including PPV, autologous retinal transplant, macular buckling, and SO tamponade.

**Outcomes:**

At 1 month postoperatively, her BCVA was improved to 20/250, and OCT showed a flattened retina with a closed macular hole. However, at 3 months postoperatively, complications such as elevated intraocular pressure, dust keratic precipitates, and anterior chamber inflammation occurred, resulting in a decrease in visual acuity to counting fingers at 1 m. These complications persisted for 1 month before stabilizing and resolving following the removal of silicone oil. Subsequent follow-up revealed stable retinal reattachment, macular hole closure, and improved BCVA to 20/100.

**Lessons:**

This combined surgical procedure resulted in improved retinal reattachment and macular hole closure, as well as the restoration of the anatomical structure and functional integrity of the retinal posterior region in the recurrent MHRD patient.

## 1. Introduction

Macular hole-induced retinal detachment (MHRD) is most commonly observed in older individuals with highly myopic eyes, and the coexistence of a posterior staphyloma leads to significant visual impairment.^[1,2]^ Parolini et al suggested that the macular detachment with full thickness macular hole should be classified as stage 4c according to the new myopic traction maculopathy (MTM) staging system.^[3]^ Several surgical techniques have been explored to enhance both anatomical and functional outcomes in cases of MHRD, including macular buckling (MB),^[4]^ pars plana vitrectomy (PPV) with internal limiting membrane (ILM) peeling^[5]^ or ILM flap inversion,^[6]^ lens capsular flap transplantation,^[7]^ autologous retinal transplantation (ART),^[8]^ human amniotic membrane grafting,^[9]^ with gas or silicone oil tamponade. Among which MB has demonstrated favorable anatomical outcomes compared to PPV alone, as it addresses the underlying posterior staphyloma and alleviates anteroposterior traction forces, resulting in improved retinal reattachment.^[10]^

According to Parolini et al,^[11]^ the proposal for the management of myopic traction maculopathy, based on the new MTM staging system, suggests that stage 4c is better treated with PPV and MB. But, for those patients who have undergone ILM peeling surgery subsequently developed retinal detachment due to macular holes face a surgical challenge because there is an inadequate amount of ILM tissue available to effectively cover and seal the retinal breaks. Simultaneously, the use of an autologous retinal flap, which is thicker and more robust, can be effectively positioned over the macular hole (MH) to provide an optimal barrier. Here we report a combined surgical technique using ART over the posterior retinal holes and MB to successfully and effectively treat recurrent MHRD.

## 2. Case report

**Figure 1.**
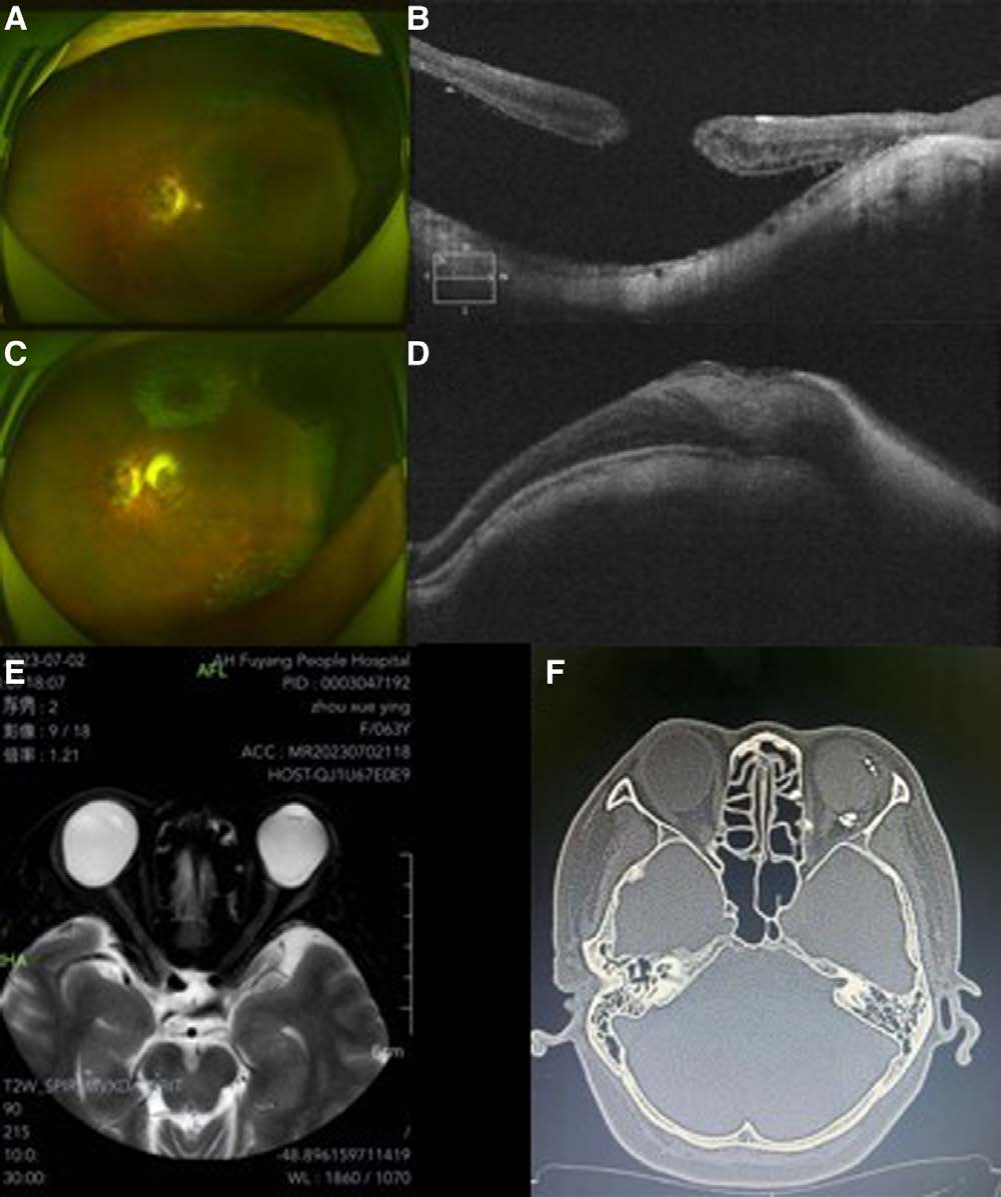
Pre- and postoperative fundus photography, OCT images, and orbital imaging of a 63-year-old woman’s left eyes. Ocular examination demonstrated retinal detachment encompassing the posterior pole with a macular hole and staphyloma (A, B). Magnetic resonance imaging (MRI) showed significant posterior bulging of the sclera (E). One week postoperative examination showed reattachment of the retina with macular hole closure (C, D). The macular buckling explant was stable and positioned away from the optic nerve (F). OCT = optical coherence tomography.

A 63-year-old female patient complained of decreased vision and central vision loss in her left eye, 2 months after the removal of silicone oil. She had previously undergone PPV, ILM peeling and filling, and silicone oil (SO) tamponade in the same eye at another hospital due to MHRD. Her visual acuity was limited to hand movement. Intraocular pressure (IOP) was 15.0 mm Hg, and the axial length (AL) was 30.81 mm. OCT and ocular examination demonstrated retinal detachment encompassing the posterior pole with a macular hole and staphyloma (Fig. 1A, B). Magnetic resonance imaging showed significant posterior bulging of the sclera (Fig. 1E). Consideration was given to the insufficiency of remnant ILM and the accessibility of the ART flap of recurrent MHRD. The patient underwent PPV, ART, MB, and SO tamponade.

The MB surgery was performed using a silicone sponge–titanium explant with retrobulbar anesthesia. The surgery began with a 120° superotemporal conjunctival peritomy, and the superior rectus and lateral rectus muscles were separated from the surrounding tissues. The rectus muscles were then looped using silk ties to allow for rotation of eye position. The explant for MB was easily prepared in the operating room with a 5 × 3 mm silicone sponge (506 silicone sponge, MIRA) and a malleable titanium plate (0.5 mm thick, 20 holes, titanium adoption plate, (Matrix MIDFACE™ Plate, Depuy Synthes, West Chester, PA). A tunnel was made into the silicone sponge with a 20-gauge microvitreoretinal blade, and the titanium plate was inserted into the tunnel.^[12]^ When the titanium plate reached the end of the silicone sponge, the tip was rolled to a snail shell shape.

Rotating the eyeball inferonasally, the explant was inserted superotemporally to the posterior of the globe to reach the macula. The buckle height was adjusted under direct visualization of the location of the buckle through the vitrectomy lens with the illuminator inserted through the trocar. The long arm of the explant was fixed to the sclera with a mattress suture posterior to the muscle insertion sites using 5–0 Dacron suture (Alcon). The remnant length of the explant was trimmed using a plate cutter and the titanium plate was cut more deeply into the silicone sponge to hide its end into the sponge.

Three-port 23-gauge PPV (Constellation; Alcon, Fort Worth, TX) was performed, and the bimanual maneuvers were used to facilitate observation of residual peripheral vitreous. Indocyanine green dye solution (25 mg indocyanine green in 20 mL 5% dextrose–water solution) was applied around the MH within the arcade to confirm the extent of the previous ILM peel and the remnants of the ILM. The site chosen for autologous retinal graft was at the superotemporal arcade, with the initial harvest area being roughly 2-disc diameters in size, and in subsequent case was calibrated according to the size of the MH.^[13]^ The edge of the graft was held, if required, using forceps (Alcon 23-g ILM forceps) and cut using vertical or curved scissors (Alcon 23-g Revolution DSP Vertical or Curved Scissors). The diathermy marks at the edges and the pattern of the retinal vessels served as anatomic markers to maintain the proper alignment of the retinal free flap. Perfluoro-n-octane heavy liquid (Perfluoron; Alcon) was instilled over the retinal flap after it was placed to cover the MH. The edges of the flap were gently flattened, and it was stretched to lay flat and cover the entirety of the hole. Subretinal fluid was gently aspirated from the peripheral retina incision site with a soft tipped needle, and the site of the peripheral retinotomy was barricaded by 2–3 rounds of endolaser. Silicone oil was slowly injected into the vitreous cavity to achieve normal intraocular pressure, parallel oil–liquid exchange, and careful removal of the perfluoro-n-octane heavy liquid.

## 3. Outcomes

### 3.1. Postoperative 1 week

The patient was recommended to lie in prone position. Fundus examination performed at postoperative 1 week revealed a bulge in the macular area associated with the local explant. The macular hole had closed, and the retina was attached (Fig. 1C, D). Her visual acuity was 20/400, and the best corrected visual acuity (BCVA) improved to 20/200. Additionally, the AL was reduced to 28.19 mm. The MB explant was stable and positioned far from the optical nerve (Fig. 1F).

### 3.2. Postoperative 1 month

**Figure 2.**
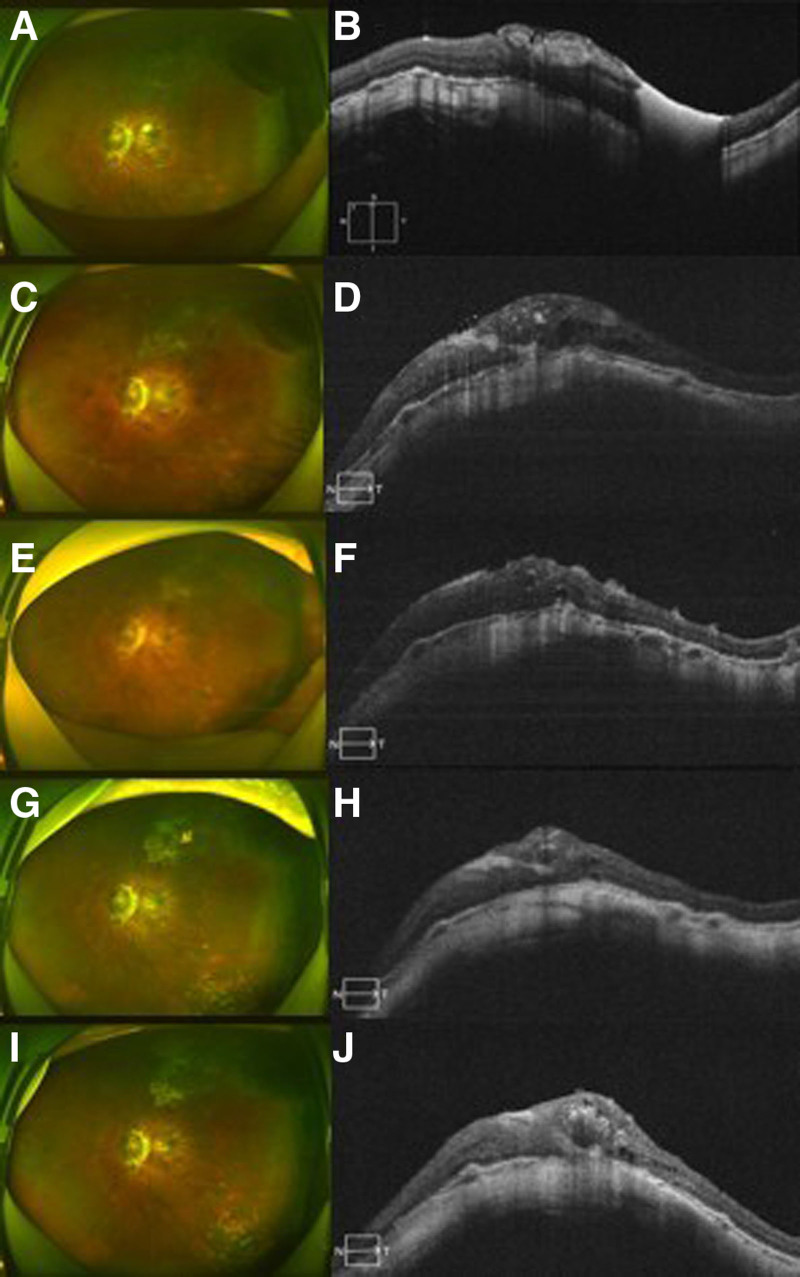
Optical coherence tomography images showing successful macular hole closure and retinal reattachment in patients. (A, B) Closed macular hole and retinal reattachment at 1 mo after combined surgery. (C, D) Closed macular hole and retinal reattachment at 3 mo after combined surgery. (E, F) Closed macular hole and retinal attachment at 1 wk after silicone oil removal. (G, H) Closed MH and retinal attachment at 1 mo after silicone oil removal. (I, J) Closed MH and retinal attachment at 6 mo after silicone oil removal.

The patient’s visual acuity improved to 20/250, and the BCVA further improved to 20/133. The AL decreased to 28.04 mm, and the IOP was 19.0 mm Hg. Fundus examination showed that the macular hole remained closed, with a bulge in the macular region and a reattached retinal. The MB explant was stable (Fig. 2A, B).

### 3.3. Postoperative 2 months

The patient’s visual acuity remained at 20/250, and the BCVA continued to improve to 20/133. The AL decreased to 28.02 mm, and the IOP was 16.0 mm Hg.

### 3.4. Postoperative 3 months

The patient experienced complications, including elevated IOP, dust keratic precipitates (KP), and inflammation in the anterior chamber, resulting in a decrease in visual acuity to counting fingers at 1 m and a blurred fundus. Topical antiglaucoma medications and steroids were administered to reduce IOP and alleviate inflammation (Fig. 2C, D). These complications persisted for 1 month with slight improvement after the application of topical medications. Given the abnormally high IOP and persistent inflammation, SO was removed via pars plana sclerotomies to prevent optical nerve damage. One week after SO removal, the cornea became slightly clear, the KP and anterior chamber inflammation were slightly relieved, and the IOP stabilized at 18.0 mm Hg (Fig. 2E, F).

### 3.5. Postoperative 1 month after SO removal (6 months postoperative of surgery)

The BCVA improved to 20/133, and the AL reduced to 26.75 mm. The elevated IOP resolved to a normal level of 16.0 mm Hg, and the anterior chamber inflammation resolved. Fundus examination showed that the macular hole had closed, and the retina was attached, indicating anatomic rehabilitation (Fig. 2G, H).

### 3.6. Postoperative 1 year

At 1 yr postoperatively, the BCVA improved and remained stable at 20/100, and the AL reduced to 26.84 mm. The IOP resolved to a normal level of 14.0 mm Hg, and the anterior chamber inflammation resolved. Fundus examination confirmed that the macular hole remained closed, and the retina was attached, achieving stable anatomic rehabilitation (Fig. 2I, J).

## 4. Discussion

In the presented case study, we described a case of treated with MB combined with ART in an eye with recurrent MHRD from the initial surgery failed. Successful retinal reattachment and macula hole close was achieved.

Traditional therapeutic strategies for MHs are extensively documented, especially PPV with peeling of the ILM and gas tamponade. While this approach is commonly used, it might not effectively manage specific underlying mechanisms, like the stress induced by posterior staphyloma, and addressing recurrent MHRD presents an even greater clinical challenge.

The MB procedure, a standard surgical approach, has witnessed the innovation of numerous explant varieties and execution techniques, such as titanium-embedded L-shaped and T-shaped silicone sponges, recognized for their convenience and affordability.^[14,15]^ Through mitigates the traction forces exerted by the posterior staphyloma and the retinal arterial tension by smoothing out the pronounced indentation at the posterior pole, thereby easing the stress on the transplanted flap’s anchorage and facilitating the drainage of subretinal fluid into the vitreous cavity. Reports suggest that among individuals with high myopia, the success rate of MH closure following PPV with ILM peeling for MHRD falls between 33.3% and 63.2%,^[16]^ whereas the closure rate post MB treatment spans from 40% to 93.3%.^[14]^ Simultaneously, the complications encountered in this case were primarily characterized by increased IOP and inflammatory responses, without the occurrence of other MB-related issues such as retinal pigment epithelial atrophic changes, extraocular muscle transection and motility limitations, optic nerve impingement, buckle exposure, or damage to the ciliary vessels and vortex veins.^[17]^

Another complexity associated with this surgical technique revolves around the sourcing of graft material. Due to insufficient ILM flap, excessive rigidity of the adjacent ILM and existed posterior staphyloma. ART is generally employed as a treatment option for MHs that are resistant to conventional surgical interventions, such as vitrectomy, ILM peeling, and gas tamponade, when reattempting the standard procedure is anticipated to yield minimal therapeutic value. Contrary to the amniotic membrane or lens capsule flaps, which involve intricate maneuvers for internal positioning within the MH and may lead to unintentional damage, the neurosensory retinal flap offers a thicker and more durable layer that can be deployed over the macular hole surface, effectively decreasing the chance of iatrogenic injury.

Peripheral retinal Müller glia could potentially serve as a reservoir for generating cells that possess the attributes of rod photoreceptors.^[18]^ The theoretical basis for ART involves the transplanted neurosensory retina serving as a scaffold for MH closure, fostering improved anatomical integration and graft revascularization through the facilitation of tissue centripetal migration from the margins of the MH.^[19]^ In addition to these structural benefits, ART might promote the process of adaptive synaptogenesis, potentially enabling the reestablishment of functional links between photoreceptor cells and adjacent bipolar or horizontal cells.^[20]^ Nevertheless, therapeutic approaches tend to separate between MB and ART, such as employing MB with traditional ILM peeling, inverted ILM peeling, PPV combined with ART. The strategy of combining MB with ILM peeling in primary MHRD surgeries has resulted in an enhanced closure rate of macular holes, reaching 82.8%, and expedited reattachment of the retina, compared to macular buckling alone, which achieved a 66.7% closure rate.^[21]^ The combined technique used for treating recurrent MHRD following unsuccessful primary surgery is rarely reported in the literature. Earlier studies report the use of lens capsule flaps or amniotic membrane in the management of retinal detachment due to retinal holes in highly myopic eyes with previous ILM peeling.^[7,9]^ However, these methods cannot solve the tensile force caused by staphyloma in highly myopic eyes. The surgical procedures employed in this study resulted in successful retinal reattachment and improved macular hole closure, while also avoiding excessive macular indentation that can lead to myriad adverse effects. This offers a safe and effective alternative for individuals with recurrent macular hole retinal detachment and insufficient ILM.

However, the combined surgical approach in this case was associated with complications such as elevated IOP, dusty KP, and inflammation response of the anterior chamber, which presented 3 months postoperatively. These complications remained stable over time and resolved following the removal of silicone oil from the vitreous cavity. Several factors have been contemplated as potential causes. Initially, inflammation of the eye is a contributing factor to increased intraocular pressure during the use of silicone oil as an endotamponade.^[22]^ Secondly, the pressure exerted by the buckling implant may disrupt the choroidal microcirculation,^[23]^ resulting in localized ischemia and hypoxia, which could subsequently lead to increased IOP and the occurrence of KP. Lastly, the use of silicone oil tamponade is a common and primary suspect for IOP elevation.^[24]^ Understanding the risk factors and pathogenesis associated with the development of high IOP after silicone oil filling can effectively prevent and reduce its occurrence, ultimately minimizing the impact on visual function.

This study has several limitations that should be acknowledged. Firstly, it was conducted as a single-site study with a small sample size, which may limit the generalizability of the findings to broader populations. Secondly, the surgical procedures described, including macular buckling and autologous retinal transplantation, may be technically challenging even for experienced surgeons. Long-term outcomes should be further evaluated through extended prospective studies to fully assess the durability and long-term benefits of this combined surgical approach.

In conclusion, combined surgery of recurrent MHRD with MB and ART achieved improved of BCVA and reattach of retina and MH closure in high myopia patients with posterior staphyloma. In addition, developing the methods to prevent further IOP elevation and anterior inflammation occurrence also seems to be important to preserve long-term postoperative visual acuity and anatomical restoration. Future prospective studies are required to provide additional evidence regarding its efficacy and safety. That combination surgery should be preferentially recommended for patients with recurrent MHRD and posterior staphyloma.

## Author contributions

**Conceptualization:** Rongrong Zhang, Mingxing Li, Jie Li, Dan Xia, Yu Li, Jiahao Li, Wenqing Deng, Chenyu Yang, Jing Wang, Jiayu Ma, Yingjun Li.

**Data curation:** Rongrong Zhang, Mingxing Li, Jie Li, Dan Xia, Yu Li, Jiahao Li, Wenqing Deng, Chenyu Yang, Jing Wang, Jiayu Ma, Yingjun Li.

**Methodology:** Rongrong Zhang, Mingxing Li, Jie Li, Dan Xia, Yu Li, Jiahao Li, Wenqing Deng, Chenyu Yang, Jing Wang, Jiayu Ma, Yingjun Li.

**Supervision:** Yingjun Li.

**Writing – original draft:** Rongrong Zhang, Mingxing Li.

**Writing – review & editing:** Rongrong Zhang, Mingxing Li, Jie Li, Dan Xia, Yu Li, Yingjun Li.
